# Hydrogel Micro-/Nanosphere Coated by a Lipid Bilayer: Preparation and Microscopic Probing

**DOI:** 10.3390/gels3010007

**Published:** 2017-02-15

**Authors:** Sarah Rahni, Sergey Kazakov

**Affiliations:** Department of Chemistry & Physical Sciences, Pace University, 861 Bedford Road, Pleasantville, NY 10570, USA; sr67443p@pace.edu

**Keywords:** lipid bilayer, lipid vesicles, hydrogels, supramolecular assembly, lipobeads, drug delivery systems

## Abstract

The result of polymeric nanogels and lipid vesicles interaction—lipobeads—can be considered as multipurpose containers for future therapeutic applications, such as targeted anticancer chemotherapy with superior tumor response and minimum side effects. In this work, micrometer sized lipobeads were synthesized by two methods: (i) mixing separately prepared microgels made of poly(*N*-isopropylacrylamide) (PNIPA) and phospholipid vesicles of micrometer or nanometer size and (ii) polymerization within the lipid vesicles. For the first time, a high vacuum scanning electron microscopy was shown to be suitable for a quick validation of the structural organization of wet lipobeads and their constituents without special sample preparation. In particular, the structural difference of microgels prepared by thermal and UV-polymerization in different solvents was revealed and three types of giant liposomes were recognized under high vacuum in conjunction with their size, composition, and method of preparation. Importantly, the substructure of the hydrogel core and multi- and unilamellar constructions of the peripheral lipid part were explicitly distinguished on the SEM images of lipobeads, justifying the spontaneous formation of a lipid bilayer on the surface of microgels and evidencing an energetically favorable structural organization of the hydrogel/lipid bilayer assembly. This key property can facilitate lipobeads’ preparation and decrease technological expenses on their scaled production. The comparison of the SEM imaging with the scanning confocal and atomic force microscopies data are also presented in the discussion.

## 1. Introduction

**Figure 1:**
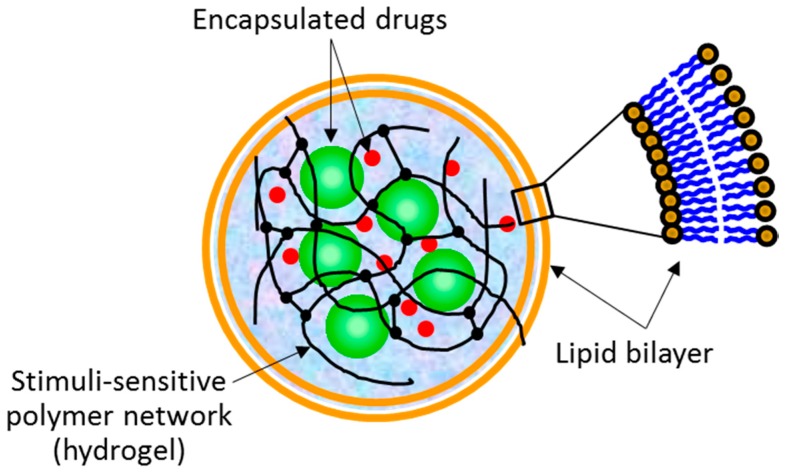
Schematic of the spherical lipid bilayer/hydrogel assemblies (lipobeads) with encapsulated drugs.

The term “lipobeads” is used to name spherical bipartite structures made of a hydrogel core coated with a lipid bilayer (Figure 1). Lipobeads (LB) belong to a class of soft matter systems, which combine the properties of synthetic but naturally related hydrogel/lipid bilayer structures. Interestingly, Nature uses properties of both a lipid bilayer and a cross-linked (physically or chemically) polymer network to provide workability, multifunctionality, and dynamism of living cells of all three main domains of life—*eubacteria*, *archaea* and *eukaryotes* [1]. In this context, synthetic hydrogels entrapped within liposomes are of special interest. For example, the sensitivity of the hydrogel core and the lipid bilayer to environmental stimuli is the foundation for practical application of lipobeads [2]. Moreover, the hydrogel/lipid bilayer structures in spherical configuration can be used for modeling molecular mechanisms of intracellular functionality and biochemical activity [3,4]. Our research group is focusing on developing lipobeads as multipurpose containers for targeted drug delivery and controlled drug release [3,4,5,6].

The concept of lipobeads as a drug delivery system promises a number of advantages at all steps of drug delivery [1], such as (i) biocompatibility and stability; (ii) capability of delivering a broad range of drugs, proteins, peptides, oligonucleotides, aptamers and so forth; (iii) variety of tiny mechanisms for controlled drug release, including consecutive multistep triggering; (iv) the potential to target specific cells within the body; (v) relevancy for systemic drug administration routes; (vi) and suitability to different diseases with possibility of efficient targeting to different organs. However, the feasibility of their production should still be estimated versus the advantages of their use as drug carriers.

The first lipid vesicles filled with hydrogel were reported in 1987, when a successful polymerization within liposomes was accomplished [7] and microspherules of agarose-gelatin filled with gold particles was encapsulated within liposomes [8]. Within 30 years of experimentation with lipobeads, it was realized that in the courses of fabrication, loading, delivery, and release, the lipid bilayers have to possess mutually opposed physicochemical properties. For example, the bilayer membrane should be thoroughly sealed to retain the inner concentrations of pre-gel components at the step of preparation and therapeutic agents at the step of delivery. On the other hand, the lipid bilayer should be sufficiently permeable to provide drug flow to the interior without losing the bilayer integrity during drug loading and drug flux to the exterior during drug release. In addition, the lipid bilayer should be stiff enough to withstand a complex environment in the bloodstream and immunological attack at the sub-organ level. Notwithstanding, it should be elastic enough at the sub-cellular level to provide lipobead trafficking to cytosol and intracellular organelles (nucleus, mitochondria, etc.). To satisfy all these contradictory requirements, the following technological aspects must be taken into account:

1. Stability and permeability of a lipid membrane can be governed by temperature and bilayer composition, because bilayers undergo a change from the liquid to the gel (solid) state at the so-called lipid (or order-disorder) phase transition temperature (*T*_t_), characteristic to the phospholipid used. In the liquid-crystalline “disordered” state, above *T*_t_, the membrane is leakier than that in the gel “ordered” state, below *T*_t_, but at the same time, it is more fluidic to favor the formation of the unilamellar membrane. It is known from the properties of naturally occurring membranes that balancing composition of cholesterol and alcohols can vary their stability and permeability. Particularly, the extent of “sealing” directly depends on the amount of cholesterol present up to moderate levels, whereas the presence of alcohol molecules in the membrane increases the membrane fluidity at a given temperature by the depression of the phospholipid order-disorder transition temperature.
2. Hydrogels presumably play a special role in the stabilization of the phospholipid bilayer. Particularly, hydrogels can promote spontaneous formation of the bilayer on their surface and can support the bilayer by enhancing its stability. In addition, the membrane permeability can be manipulated by physical contraction/expansion of the environmentally sensitive hydrogel supporting the bilayer [9].
3. Properties, functionality, and application areas of lipobeads depend on their production methods. Two methods are available to date for the preparation of artificial bilayer-coated hydrogel particles (see [1] and references therein). The first one employs the liposomal interior as a chemical reactor for the formation of hydrogels by polymerization. The second one is based on the formation of lipid layers around hydrogels after microgel-liposome mixing.

Our main goal is to study the compatibility of hydrogels and lipid bilayers, since it might be this property that could reduce the cost of lipobead production. We report here the synthesis of lipobeads with temperature-sensitive hydrogel cores made of poly(*N*-isopropylacrylamide) (PNIPA) using two methods: polymerization within giant vesicle interiors and mixing separately prepared microgels and phospholipid vesicles of micrometer (giant vesicles) or nanometer (liposomes) size. Depending on the size, lipobeads can be classified into two groups: nanolipobeads (NLB, <1000 nm) and giant lipobeads (GLB, >1 µm). Although the nanolipobeads of ~100-nm size are the most relevant objects for the development of realistic drug delivery systems [10], in this paper we use the giant lipobeads as a model for direct observation of the hydrogel/lipid bilayer structural features under optical, fluorescence and confocal microscopes. To visualize the fine structure of nanolipobeads or the substructure of giant lipobeads, one may also consider Scanning Electron Microscopy (SEM), a powerful research technique with the resolution higher than the diffraction limit of optical microscopy.

Usually, due to high vacuum requirements in the sample chamber, the wet soft materials, like biological samples, hydrogels and liposomes [11,12], undergo several rigorous sample preparation steps to dehydrate the sample (fixation, staining, freeze-fracture). Moreover, the samples should be prevented from charging under the imaging electron beam (sputter metal coating). These drawbacks were overcome by using the environmental SEM (ESEM), which operates in a gaseous environment [11,12,13]. Introduction of gases into the imaging chamber at a relatively high pressure (10–3000 Pa) allows for visualization of hydrated soft specimens to be performed without conducting coatings. Actually, ionization of the gaseous environment prevents the surface charging of nonconductive samples under the microscopic study. Furthermore, in March 2016, the Hitachi High-Technologies Corporation introduced [14] a novel atmospheric SEM, “Aerosurf 1500”, for observation of wet samples at atmospheric pressure (10^5^ Pa) without additional preparations. Nevertheless, the advent of environmental and atmospheric SEMs should not be overestimated, since one has to expect a decrease in resolution due to collisions of electrons with the chamber atmosphere.

So, it was traditionally thought that it would be impossible to study hydrated samples by the high vacuum SEM, because the sample dehydration could cause significant destructions of its soft and fragile structural features. However, we envisioned that it is the softness of hydrogels, liposomes, and eventually lipobeads that could allow these hydrated objects to withstand high vacuum in the imaging chamber. Thus, in the first part, we aim at demonstrating that the external layers of the aforementioned particles collapse under high vacuum, sealing the water within the structure, so that a high vacuum SEM can be used to reveal the difference in structures of hydrogels prepared by thermal and UV polymerization in different solvents and to compare the conventional method for giant vesicles preparation, based on lipid film gentle hydration, with the injection of an ethanol solution of lipids into hot water. In the second part, we test the formulations of different lipidic composition in terms of their structures and interactions with the surface of microgels, namely: two phospholipids with the order-disorder phase transition temperatures (*T*_t_) below and above room temperature, with or without cholesterol. Also, we examine the lipobeads prepared by the two aforementioned methods. Herein, we would like to test whether a spherical microgel can be covered by a lipid bilayer even if it is mixed with multilamellar vesicles. The SEM probing of lipobeads is compared with the scanning confocal microscopy data in the discussion section. For characterization of lipobeads under the laser scanning confocal microscope, fluorescent monomer (FA) co-polymerized with polymer network and fluorescent phospholipid (RhodB-PE) are used as fluorescent labels for the hydrogel core and the lipid bilayer, respectively.

## 2. Results

### 2.1. PNIPA-FA Hydrogels under High Vacuum

#### 2.1.1. Bulk Hydrogels Prepared by Different Methods

To examine if the traditional SEM imaging can resolve (despite the deformations under high vacuum) structural distinctions of the 3D-polymer networks prepared by different methods, three bulk hydrogels were synthesized, namely: thermal polymerization in water or dimethyl sulfoxide (DMSO) as solvents, and UV polymerization in water (see Materials and Methods section for details).

**Figure 2:**
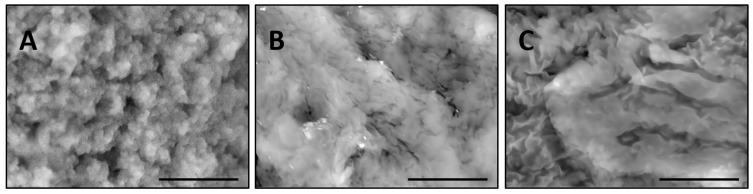
Comparison of the microstructures of bulk wet PNIPA-FA hydrogels prepared by thermal polymerization in water (**A**) or DMSO (**B**) and by UV polymerization in water (**C**) (scale bars = 5 µm).

Figure 2 represents the corresponding microstructures obtained by SEM without special sample preparation. Thermal polymerization in water yields a granular structure (Figure 2A) with the cross-linked domains and pores of submicrometer sizes. On the contrary, the analogous polymerization in DMSO yields a denser structure with nanoscaled features (Figure 2B). This difference can be explained by an opposite liquid-liquid phase-diagram behavior of PNIPA in water and DMSO [15,16]. A water/PNIPA system exhibits a Lower Critical Solution Temperature (LCST) behavior, as opposed to DMSO/PNIPA, meaning that thermal polymerization and cross-linking of PNIPA occur at temperatures when polymer forms in a shrunken state in water and in a swollen state in DMSO.

Photopolymerization in water also leads to a denser structure (Figure 2C) in comparison with thermal polymerization in water (cf. Figure 2A), but structural peculiarities are different in comparison with thermal polymerization in DMSO (cf. Figure 2B). The latter structure could be called “striped”, as it comprises the stripes cross-linked at the submicroscopic level.

**Figure 3:**
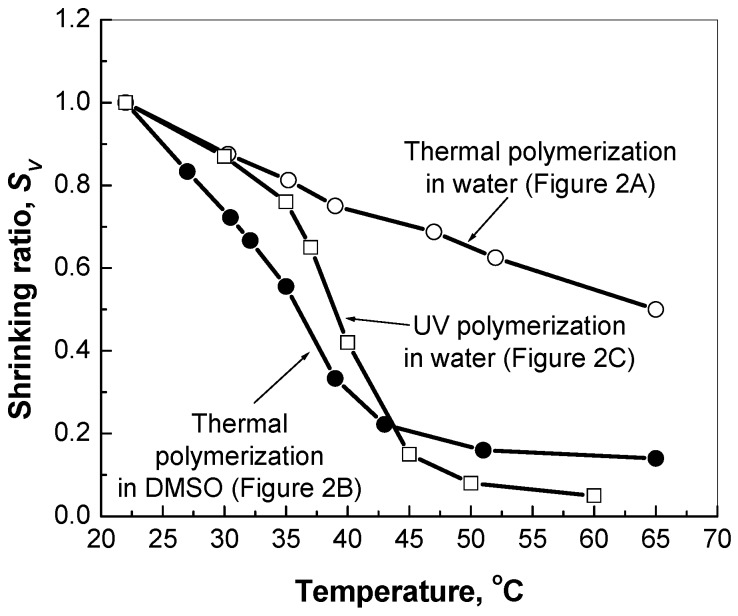
Shrinking abilities *S_V_* of the bulk PNIPA-FA hydrogels prepared by thermal and UV polymerization in different solvents.

Interestingly, the granular structure of the PNIPA-FA hydrogels can be broken down into separate submicroscopic domains by means of sonication (data not shown). On the contrary, sonication does not affect the structure of “dense” hydrogels. Figure 3 shows that the temperature-sensitive shrinking ability of hydrogels depends on their microstructure: the granular hydrogel exhibits a continuous volume decrease with temperature, whereas the denser hydrogels exhibit more abrupt changes in volume within the range of temperatures from 35 to 45 °C.

#### 2.1.2. PNIPA-FA Microgels under High Vacuum

**Figure 4:**
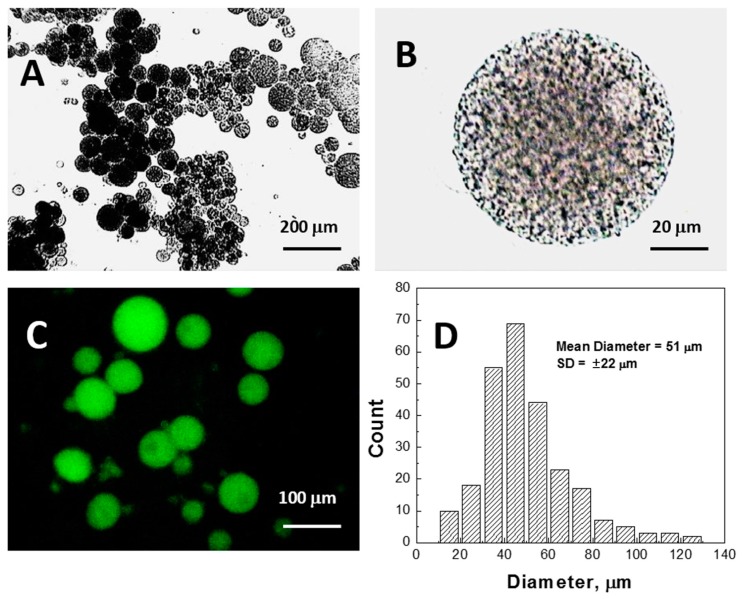
The bright field (**A**,**B**) and fluorescence (**C**) images and size distribution (**D**) of PNIPA-FA hydrogel spheres after washing.

The size distribution of PNIPA-FA microgels prepared by thermal ISP method was examined by measuring approximately 300 particles using the image analysis software (Motic Image 2.0 Plus, Motic Instruments, Inc., Richmond, BC, Canada). Optical micrographs (e.g., Figure 4A) and image analysis (Figure 4D) indicate that the diameter of the microgels range from 10 to 130 µm. It was found that the microgels were very sticky and had a porous fine substructure (Figure 4B). Fluorescence microscopy (Figure 4C) explicitly shows the homogeneous distribution of fluorescent monomer (FA) covalently attached to the PNIPA network.

**Figure 5:**
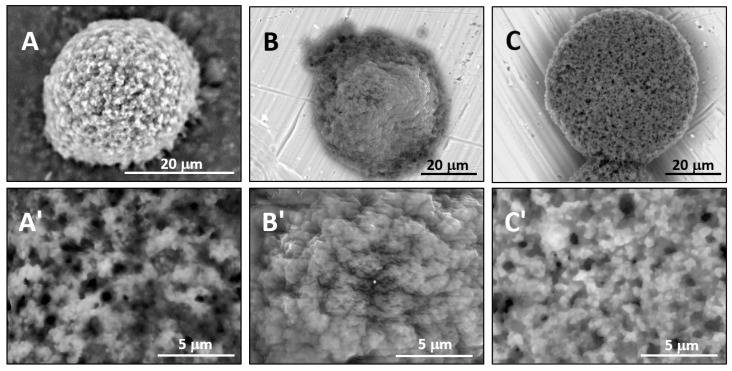
SEM micrographs of the PNIPA-FA hydrogel spheres filled with water (**A**,**B**) or ionic liquid (**C**) and deposited on the carbon (**A**) or aluminum (**B**,**C**) substrates of the SEM mount.

To get an insight into the microgel structure, we use the SEM. Unexpectedly, these wet soft microgels were able to withstand the high vacuum involved with SEM. Figure 5A,B represent the structure of the microgels deposited on the carbon and aluminum surfaces, respectively. In both cases, under high vacuum, the particles collapsed trapping water inside. However, it appears that the mechanism of collapsing depends on the adhesion surface. One can visualize the microgel preserving a spherical shape without flattening on the carbon surface (Figure 5A), probably due to decreased wetting. On the contrary, on the aluminum surface (Figure 5B), the microgel takes a hat-like shape with a flat peripheral area and a central part filled with water, probably due to a higher extent of wetting. Different brightness of these areas on the backscattering electron (BSE) micrographs corresponds to the surface charge distribution. To increase the sample conductivity, we replaced water inside microgels with ionic liquid (HILEM IL 1000) [17] by immersing the microgels in a 10% IL 1000 aqueous solution for 10 min followed by absorption of the excess solution with a strip of filter paper. Figure 5C shows that there is no surface charging, but the hydrogel particle becomes uniformly flattened on the aluminum surface. Importantly, a granular microstructure of the wet microgels filled with either water or ionic liquid can be recognized on the high vacuum SEM images under higher magnifications. Three structural features should be highlighted: (i) the microgel structure consists of cross-linked domains smaller than 1 µm (Figure 5A′, ×10k); (ii) in the course of the external layer collapsing, the domains sinter to form a dense crust, which seals the water inside the particle (Figure 5B′, ×10k); and (iii) the domains seem to shrink within a microgel filled with ionic liquid (Figure 5C′, ×10k). Actually, since the hydrogel is capable of swelling or shrinking in the environment of ionic liquid [18], further study of the effect of ionic liquid on the shape and size of PNIPA hydrogel is in demand. All particles studied in this work were filled with water, if not otherwise stated.

### 2.2. Giant Vesicles

Giant multilamellar vesicles (GMV) were prepared by two methods: (i) gentle lipid film hydration and (ii) injection of a phospholipid/ethanol solution into hot water. The lipidic formulations contained either HSPC (*T*_t_ = 52 °C) or EPC (*T*_t_ ≤ 0 °C) with or without cholesterol as described in Section 4.1. The fluorescent phospholipid (RhodB-PE) was added to visualize the formed lipid bilayers under confocal microscopy. Six lipidic formulations studied by SEM are systemized in Table 1. Notice that formulation 1 represents the giant multilamellar vesicles with the most solid lipid bilayer made of the phospholipid with the order-disorder phase transition temperature (*T*_t_) much higher than room temperature and containing cholesterol, whereas formulation #6 represents the GMVs with the most fluidic lipid bilayer prepared by hydration of the lipid film consisting of the phospholipid with *T*_t_ lower than room temperature and no cholesterol added.

According to the analysis of the GMVs using Confocal Laser Scanning Microscopy (CLSM) (the data not shown), multilamellar vesicles with the size ranged from 1 to 30 µm were formed in all cases. The straightforward observation was that fluidity of the lipid bilayer is a critical parameter for the final morphology of the vesicles. Indeed, vesicles made of the phospholipid with a higher *T*_t_ (HSPC) and cholesterol did not fuse feasibly, whereas the phospholipids with a lower *T*_t_ (EPC) and the absence of cholesterol promoted the formation of more homogeneous lipid bilayers, probably due to favorable fusion. Moreover, suspensions of vesicles prepared by gentle hydration looked more homogeneous with a bigger portion of unilamillar vesosomes, in comparison to the ones prepared by the “injection” method.

Columns 2–4 in Table 1 show BSE micrographs of the wet vesicles for all six lipidic formulations. What appears to be a different topology of the giant vesicles deformed under high vacuum can be observed on the surface of the SEM mount. Depending on the size, three types of the vesicles were distinguished.

The particles smaller than ~5 µm collapsed into smooth spheres containing water inside (Table 1, column 2). Explicitly visible dark halos around the vesicles on the aluminum surfaces were reproducible and most likely can be assigned to the portion of a vesicle from which water was evaporated under high vacuum. The remaining lipid bilayers squeeze to form a tight dense layer, which protects water inside from further evaporation. A retaining of the spherical shapes and formation of the water sealing layers are probably possible due to a high curvature of the small vesicles resulting in the layer surface tension increase.

The vesicles of medium size (~5–20 µm) collapsed into near spherical structures with rippled surfaces since their surface area is getting too large to retain smoothness (Table 1, column 3). These particles are also surrounded by the dark halos, and sealed enough to hold water inside the central ­part. Moreover, some of them are multivesiclular, i.e., vesosomes containing smaller vesicles inside (see, for example, formulations 5 and 6).

The vesicles greater than 20 µm in diameter (Table 1, column 4) are mostly multilamellar and/or vesosomal per se. Under high vacuum, they collapse on the SEM mounts to form the flattened structures with explicitly recognized internal vesicles. Wrinkled surfaces of the giant vesicles prepared by gentle hydration (formulations 5 and 6) are typical for the lyophilized giant liposomes.

### 2.3. Lipobeads

#### 2.3.1. Mixing of Microgels and GMVs

Six lipidic formulations were mixed with the microgel suspensions and incubated overnight with three freezing/thawing (for EPC) or heating/cooling (for HSPC) cycles. After washing by centrifugation, the giant lipobeads (GLBs) were visualized using the high vacuum electron microscopy without drying and metal coating. The results were systemized in Table 2.

At first glance, it is evident that microgels have lipid coats in all cases. Besides the lipid bilayer coating, many unfused vesicles adsorbed onto the surface of microgels are apparent as well. Therefore, one can conclude that it is virtually difficult to separate the lipobeads from unfused and unbound GMVs, since their sizes and densities are similar. On the other hand, the greater amount of lipidic structures left around a microgel (see for example formulations 2, 3 and 6), the less flattened and more spherical is the shape of a lipobead, since it retains more water in its central part. These features can be also recognized by the different brightness of central (more charged) and peripheral (less charged) parts on the BSE images of GLBs presented.

#### 2.3.2. GLBs by Gelation within Giant Vesicles

**Figure 6:**
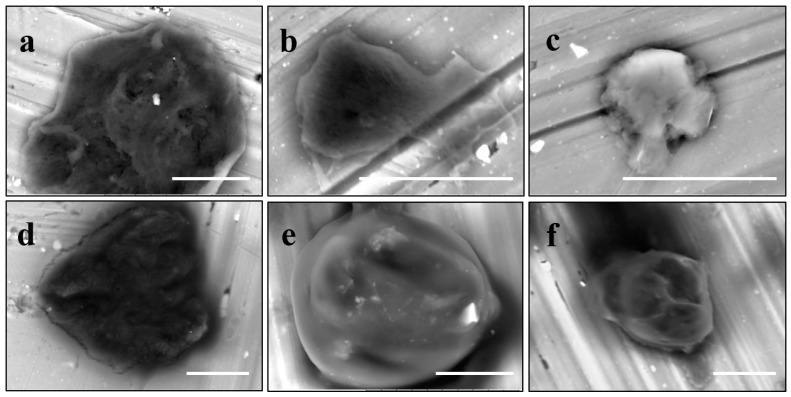
SEM micrographs of the wet lipobeads prepared by polymerization inside GMVs (**a**–**c**) or giant unilamellar vesicles (GUVs) (**d**–**f**) and deposited on the aluminum SEM mounting stub (scale bars = 10 µm).

After polymerization within giant vesicles, the washed GLBs were visualized using the standard SEM. Figure 6 demonstrates a diversity of the generated wet lipobeads placed into the high vacuum chamber without additional sample preparation. Although, in general, each particle has a central polymeric part and a peripheral lipidic part flattened during deposition on the SEM mount, one can notice the lipobeads of various size and morphology, e.g. irregular mixture of polymeric and lipidic multilayered networks (Figure 6a); irregular polymeric network within a unilamellar external lipid bilayer, which looks like a spill on the mounting stub surface (Figure 6b); a number of lipobeads tightly coalesced into one particle (Figure 6c); and near-unilamellar lipobeads with well-recognized a flattened lipidic portion and non-spherical (Figure 6d) or nearly spherical (Figure 6e,f) polymeric contents.

**Figure 7:**
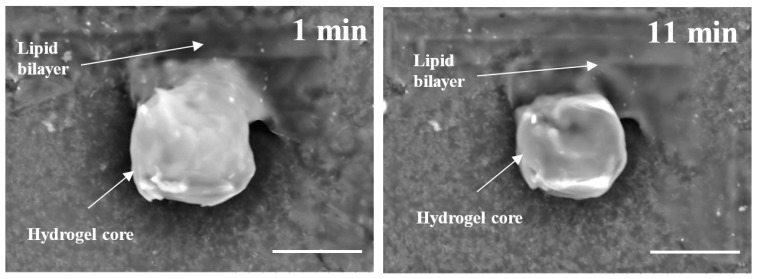
The temporal changes in a lipobead morphology under the scanning electron beam (15 kV) in high vacuum (~50 Pa). The lipobead is prepared by polymerization inside a GUV and deposited on the carbon conductive tab preliminary adhered on the SEM mounting stub (scale bars = 20 µm).

Strikingly, we were able to visualize dynamic changes that occur to lipobeads under continuous exposure to the electron beam. For example, the BSE image of a lipobead prepared by polymerization within a giant unilamellar vesicle (GUV) taken after 1 min under the scanning electron beam is presented in Figure 7 (left). Deposition of a lipobead on the carbon substrate provides a stark contrast between the central microgel and peripheral lipid bilayer, whose surface area is large enough not only to cover the microgel, but also to flatten aside like a spill. After 11 min under the scanning electron beam (Figure 7, right), the lipid coating melts, whereas the polymeric part of the lipobead shrinks. The dynamic nature of these changes cannot be achieved with the traditional sample preparations, such as freeze-fracture or/and conducting metal coating [13].

## 3. Discussion and Conclusions

### 3.1. Microgels on Carbon and Aluminium Surfaces of SEM Mount

One of the most interesting findings of this work is that the SEM imaging provides significant information in regards to the morphology and structural features of the wet microgels, lipid vesicles, and lipobeads with little preparatory work. The results demonstrate that the external layers of the aforementioned particles collapse under high vacuum, sealing the water within the structure. It was discovered as well that the same wet samples could change in appearance, however, if they are settled on different surfaces and filled with different liquids. Figure 5 compares PNIPA-FA microgels from the same batch prepared by inverse suspension polymerization.

When a microgel filled with water is placed down on a conductive carbon tag (Figure 5A), it retains a near-spherical shape and fine structure of the polymer network. By contrast, the same wet microgel deposited on an aluminum mounting surface (Figure 5B) partially spreads to take a hat-like shape with the compressed external polymeric layer sealing the water in the central part of the particle. Remarkably, when a microgel is filled with a 10% ionic liquid solution, it completely spreads on the aluminum surface. In actuality, one can recognize three regimes of wetting on these images: incomplete wetting (Figure 5A), when the contact (wetting) angle θ ≥ 90°; good wetting (Figure 5B), when the liquid has a strong affinity for the solid and θ < 90°; and total wetting (spreading) (Figure 5C), when θ = 0°.

These results indicate that further research on the interfacial behavior of not just liquid drops but the drops encapsulated into 3D polymeric networks or within closed lipid bilayers is in demand. For example, the conductive adhesive is a carbon-filled acrylic, free of solvents, so that it is unknown whether the wetting behavior results from the hydrophobicity of carbon or acrylic glue. Thus, in order to explain the spreading in Figure 5C, one should address not only the liquid/surface interactions, but also the polymer matrix/surface and liquid/polymer matrix ones.

### 3.2. Confocal Microscopy, Atomic Force Microscopy, and Atmospheric SEM

It is interesting to compare the high vacuum SEM imaging of lipobeads with the other microscopies, which also offer scenarios for visualization of the soft matter systems in their hydrated state without fixing, staining, freezing, coating or other sample preparatory work. Figure 8A shows that confocal and SEM microscopies are complementary in revealing both the unilamellar lipid layer around microgels and unfused vesicles adsorbed onto the surface of microgels. Figure 8B confirms the formation a homogeneous lipid layer around microgel, when the large unilamellar vesicles (prepared by a long-term sonication of GMV suspension) are incubated with microgels. Herein, the smaller unfused vesicles are readily washed out from the lipobead suspension by low-speed centrifugation. Sample preparation for fluorescent and confocal microscopies is the same as for optical microscopy, so that these techniques are suitable for viewing the samples in a fully hydrated state, even in solution. However, they are limited to the samples of the micrometer size-scale. Additionally, for confocal microscopy, more time is required for setting the parameters (rate of scanning) and focusing of the image to obviate possible bleaching.

**Figure 9:**
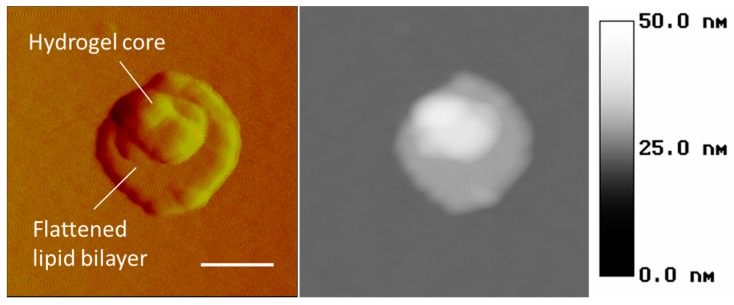
Atomic force microscopy images (amplitude and height data) of a PNIPA nanolipobead resulted from polymerization within a large unilamellar vesicle (LUV) (scale bar = 100 nm, right insert is the height scale). The lipobead was deposited on mica as described in Section 4.

Atomic force microscopy (AFM) is the other method for probing the wet particles on the nanometer scale. In particular, Figure 9 demonstrates the capability of this technique to recognize the lipid bilayer as the peripheral flattened part and the hydrogel core as the central bulging part of a nanolipobead. It is obvious that the topology of liposomes, nanogels, and nanolipobeads under AFM depends on their interaction with the surface of mica, which the samples are typically deposited on.

Atmospheric SEM (AeroSurf, Hitachi, Japan) is a novel technique allowing wet systems to be viewed under atmospheric conditions. The presence of vapor in the sample chamber is made possible by a combination of the pumping zones with high vacuum for the electron gun and partial vacuum or atmospheric pressure in the sample chamber. The expected advantages of this technique are amplification of the secondary electron signal and prevention of sample changing due to ionization/deionization of gaseous molecules. However, it was observed that the resolution of imaging the samples studied in this paper dropped drastically at near-atmospheric pressures in comparison with the one taken at high vacuum (data not shown).

### 3.3. Closing Remarks

There is no ideal technique for visualization of wet materials, and all types of microscopy have their advantages and disadvantages in terms of resolution, sample preparation, measuring conditions, and interpretation of artifacts. Within this paper, we have discovered a capability of the traditional high vacuum SEM for quick characterization of soft polymeric networks, uni- and multilamellar lipidic vesicles, and lipobeads in their hydrated states.

Firstly, the SEM images have revealed various structural features of polymeric networks synthesized by the inverse suspension polymerization in different solvents and using different initiators. It was found that the PNIPA-FA micro- and macrogels prepared by photopolymerization in water or thermal polymerization in DMSO had a “denser” and stronger structure than the granular ones prepared by the thermal polymerization in aqueous medium. Herein, sonication disintegrated the hydrogels with granular structure on separate domains of micrometer size, whereas the “dense” structure of a stronger hydrogel is not affected by sonication. Moreover, the stronger PNIPA hydrogels exhibited more pronounced volume change with temperature elevation in comparison to the granular hydrogels prepared in water. These findings will allow us to design drug delivery systems with different drug release profiles.

Secondly, it was shown that the traditional high vacuum SEM remains a useful tool for investigation of structural characteristics of soft lipidic formulations such as submicrometer sized liposomes and giant multilamellar vesicles prepared by different methods. In particular, the observed smooth spherical particles containing water inside were assigned to the small liposomes (<5 µm), the near-spherical structures with rippled surfaces were assigned to the vesicles of medium sizes ranged from 5 to 10 µm, and the vesicles bigger than 10 µm, which formed depending on the preparation method either flattened structures with explicitly recognized internal multilamellar vesicles or structures with wrinkled surfaces typical for the lyophilized giant liposomes. The results indicate that stability and elasticity of lipid bilayers, which are crucial for the lipobeads preparation, could be quickly validated for different lipid compositions and methods of preparation.

Thirdly, it has been proven that even under high vacuum, SEM imaging was able to confirm the structural organization of lipobeads in their hydrated state with little preparation work. Indeed, substructures of the polymeric central part and multi- and unilamellar constructions of the peripheral lipid part were explicitly distinguished on the SEM images of lipobeads prepared either by mixing of microgels with lipidic formulations or by polymerization within giant vesicles. The SEM observations of spontaneous formation of the bilayer on the surface of microgels justify compatibility of hydrogels and phospholipid bilayers for all studied lipidic formulations and provide evidence for the hydrogel/lipid bilayer as an energetically favorable structure.

The other important finding is that besides lipid coating, one can notice many unfused vesicles adsorbed onto the surface of microgels when mixed with GMVs. Keeping in mind that six lipidic formulations represent GMVs with different multilamellarity and stiffness, one can conclude that those are the factors that make GMVs virtually difficult to fuse on the surface of microgels, and that the unfused and unbound vesicles cannot be separated from lipobeads, because of their similar size and density. Both SEM imaging and confocal microscopy showed that the small (sonicated) vesicles spontaneously fused to form a homogeneous lipid layer around microgel spheres, whereas unfused vesicles can be readily washed out from the lipobead suspension by a low-speed centrifugation.

As outlined in our recent review [1], thus formed lipobeads not only retain all the important benefits of polymeric and liposomal drug carriers in one construct [19], but also bring new superior properties [20] and provide a number of novel and unique drug delivery options [21]. The results of this work validate that technological expenses on the production of lipobeads would not be a high cost for the gained advantages of their use: (i) the major methods for lipobeads’ synthesis (polymerization within liposomal interior and liposome/hydrogel mixing) and for drug loading (polymerization in the course of hydrogel core preparation and soaking the dry hydrogel particles in a drug-dissolved solution) are analogous to those of conventional liposomes and nanogels; (ii) the lipid vesicles are compatible with microgels to form lipid coats around them spontaneously; (iii) the injection of phospholipid/ethanol solution into hot water could be used as an alternate method for the preparation of lipidic vesicles without time-consuming steps of lipid film formation and hydration (the time for the scaled fabrication of lipobeads can be reduced from days to hours).

Our future research will focus on the release of drug-imitated molecules from the lipobeads in the course of hydrogel core swelling/shrinking within the range of physiological temperatures.

## 4. Materials and Methods

### 4.1. Materials and Chemicals

Lipids used in this work are Egg PC l-α-phosphatidylcholine (*T*_t_ ≤ 0 °C, EPC), Hydro Soy PC l-α-phosphatidylcholine (*T*_t_ = 52 °C, HSPC), cholesterol (Chol), 1,2-dipalmitoyl-*sn*-glycero-3-phosphoethanolamine-*N*-(lissamine rhodamine B sulfonyl) (ammonium salt) (16:0 Liss RhodB-PE, λ_Ex_/λ_Em_ = 560/583 nm) from Avanti Polar Lipids (Alabaster, AL, USA). *N*-isopropylacrylamide (NIPA) as a monomer, *N*,*N′*-methylene-*bis*-acrylamide (MBA) as a cross linker, fluorescein-*o*-acrylate (FA, λ_Ex_/λ_Em_ = 490/520 nm) as a fluorescent monomer, ammonium persulfate (APS) as an initiator, *N*,*N,N′*,*N′*-tetramethylenediamine (TEMED) as an accelerator, sorbitane monostearate (Span 60) as a surfactant, 2,2-diethoxyacetophenone (DEAP) as a photoinitiator, and solvents (dimethyl sulfoxide, cyclohexane, chloroform, methanol, and ethanol of chemical grade) are purchased from Sigma Aldrich (Saint Louis, MO, USA). Water purified by RiOs-16 Essential Water Purification System (EMD Millipore, Billerica, MA, USA) at the resistivity of 16 MΩ·cm was used in all experiments. All chemicals are used as purchased without further purification.

### 4.2. Preparation of Bulk Hydrogels

The poly(*N*-isopropylacrylamide) hydrogel (PNIPA) was prepared either by thermal polymerization or photopolymerization. The composition of hydrogel-forming solutions (HGFS) and conditions of polymerization are summarized in Table 3.

The procedure of UV-induced polymerization used DEAP as a photoinitiator. A Blak Ray mercury lamp (365 nm, 100 W) was a UV light source to initiate polymerization. This polymerization occurred at room temperature, i.e., below the lower critical volume phase transition temperature (LCST) for PNIPA (*T*_v_ ~ 32–37 °C).

In the procedure of thermal polymerization, APS was used as an initiator and TEMED as an accelerator. For the thermal polymerization, bulk hydrogels were synthesized at 60 °C in two different solvents: water and dimethyl sulfoxide (DMSO).

Swelling/shrinking ability of the bulk hydrogel prepared was measured by the so-called method of “precipitated cylinder” [22]. The macrogels were washed with a large amount of deionized water, extruded through a metallic membrane with 160 μm pores. For every type of the hydrogels, a certain amount of thus prepared microparticles was suspended in 2.6 mL of water within cylindrical vial of inner ∅ 12 mm. Suspension was sonicated for 10 min, then the microgel particles precipitated forming a milky cylinder. After establishing an equilibrium (~2 h) at a certain temperature, the height of the cylinder in the vial was measured, so that the shrinking ratio *S*_V_ = *h/h*_22_ was calculated, here *h*_22_ is the height in a state at 22 °C, *h* is the height in a state at a higher temperature.

### 4.3. Preparation of Giant Vesicles and Liposomes

#### 4.3.1. Gentle Hydration Method

A needed volume of phospholipid/cholesterol (9:1 molar ratio) solution with total lipid concentration of 7.1 mM in chloroform/methanol (9:1 *v*/*v*) was poured into a round-bottom flask. Rhod B-PE was added (5 µM) to provide fluorescent staining of the lipid bilayer for the confocal microscopy imaging. To form a lipid film (cake), the chloroform was evaporated under flushing with nitrogen. The film was held under vacuum overnight and then hydrated by dispersing in distilled water (or HGFS) at a temperature higher than *T*_t_ characteristic to the phospholipid used. The final concentration of a phospholipid was chosen to be 5 mg/mL. To form giant vesicles with a diameter >1 µm, the steps of rough agitation were excluded from the procedure, so that the sample was incubated overnight in a water bath following by 3–5 freezing/thawing cycles for EPC.

#### 4.3.2. Injection of the Ethanol Solution of Phospholipids into Hot Water Method

The modified procedure from [23] was used. Briefly, bilayer-forming lipids (phospholipid, cholesterol, Rhod B-PE) were dissolved in ethanol to the total concentration of 0.45 g/mL. This solution was injected into hot water to make the final concentration of 5 weight % of ethanol. The temperature of the water was kept above the phase transition temperature (*T*_t_) specific for the phospholipid used. After complete dispersion of lipid, the formulation was left overnight and a series of freezing/thawing (for EPC) or heating/cooling (for HSPC) cycles were imposed in order to form a dispersion of giant multilamellar vesicles (GMV). Similar to the gentle hydration method, the composition of the formulations was calculated based on the total concentration of phospholipids of 5 mg/mL and the final fluorescent phospholipid concentration of 5 µM. Different lipidic formulations were prepared using two types of phospholipids (HSPC or EPC). If cholesterol was added, the phospholipid/cholesterol molar ratio was 9:1.

Whatever the procedure of GMVs generation was, if the large unilamellar vesicles (LUVs, <1 µm) were needed, the suspension of giant vesicles was subjected to a long-time (2 h) sonication (Ultrasonic Cleaner 50D, VWR International, Radnor, PA, USA).

### 4.4. Gelation within Giant Vesicles

To prepare giant vesicles filled with a hydrogel forming solution (HGFS), a mixture of monomers (5.5 wt % of NIPA, 0.02 wt % of FA), a cross-linker (0.5 wt % of MBA), and a photoinitiator (0.1 wt % of DEAP) in distilled water was used at the step of lipid film hydration. After overnight incubation and freeze/thaw cycles, the contents of the interior and exterior of giant vesicles was supposed to be identical. To prevent polymerization in the solution exterior to the giant vesicles, the resultant suspension was diluted 20-fold with distilled water. Immediate UV exposure (Blak Ray mercury lamp, 100 W, UVP, LLC, Upland, CA, USA) of the dispersion initiates free radical polymerization interior to the giant liposomes. After 1 h of polymerization at 25 °C, the dispersion was centrifuged for 15 min at 3100 rpm (IEC Medilite Microcentrifuge, Thermo Fisher Scientific, Waltham, MA, USA); water was replaced followed by 30 min of soaking. This washing process was repeated five times to ensure that the lipobeads were free from unreacted chemicals.

### 4.5. Microgel Preparation

The poly(*N*-isopropylacrylamide-*co*-fluorescein-*o*-acrylate) (PNIPA-FA) microgels with fluorescence ability were prepared using an inverse suspension polymerization (ISP) method. The oil phase consisted of cyclohexane (40 mL) and Span 60 (11.6 mM) as an oil soluble surfactant was sonicated for 10 min and purged with nitrogen for 2 min in a 250 mL two-neck round-bottom flask. A 4-mL aqueous phase containing NIPA monomer (725 mM), MBA cross-linker (72.4 mM), and FA fluorescent monomer (86 µM), and APS initiator (12.4 mM) was injected dropwise into the oil phase under continuous 800 rpm–stirring and N_2_–purging. After 30 min of stirring, the polymerization was accelerated by adding TEMED (8.3 mM). Stirring was stopped after 10 min, and the suspension was placed in a water bath at 40 °C and left for 24 h to complete polymerization. The top layer of cyclohexane was removed, and the lower layer was dissolved in 40 mL of ethanol and transferred to the 50-mL centrifuge tube. After 2 h of soaking in ethanol, the suspension was centrifuged for 15 min at 6000 rpm (Durafuge 200, Precision, Winchester, VA, USA). After washing three times in ethanol, the procedure was repeated three times in water to ensure that all microgels dispersed in aqueous medium were free of unreacted chemicals.

### 4.6. Giant Lipobeads by Microgel/GMV Mixing

Giant lipobeads were prepared by mixing 0.250 mL of microgel suspension with 0.250 mL of 5 mg/mL of six lipidic formulations from Table 1. The preparations were incubated overnight followed by three freeze/thaw (for EPC) or heat/cool (for HSPC) cycles. The lipobeads were washed up to three times in distilled water by centrifugation for 15 min at 3100 rpm.

### 4.7. Instrumentation

#### 4.7.1. Optical Microscopy (OM) Imaging of Microgels

A National Optical Compound Phase Contrast Digital microscope (DC3-163-PH, Microscope World, Carlsbad, CA, USA) is used to observe and estimate the size, shape, and morphology of microgels and giant lipobeads. The microscope is equipped with a CCD camera with integrated imaging system (Motic Image Plus 2.0, Motic Instruments Inc.).

#### 4.7.2. Confocal Laser Scanning Microscopy (CLSM) Imaging of Lipobeads

A confocal laser scanning microscope (LSM 700, Carl Zeiss, Inc., Thornwood, NY, USA) is used to image optical sections of microgels covered by a lipid bilayer. To distinguish the compartments of lipobeads on dual-color confocal images, two fluorescent probes are used. The green image of the hydrogel core originates from fluorescein-*o*-acrylate covalently attached to the PNIPA network. The red image of the lipid bilayer originates from Rhod B covalently attached to the heads of phospholipids.

The samples for optical and confocal microscopy are prepared by placing an aliquot of a suspension between a 2-propanol cleaned depression glass slide (40 µL) and No. 1 coverslip. To prevent the evaporation of water during observation, the sample is sealed along the perimeter of the coverslip by a nail polisher.

#### 4.7.3. SEM Probing of Microgels, Lipid Vesicles, and Lipobeads

Backscattering electron (BSE) micrographs are obtained using a Hitachi Tabletop Microscope TM3030Plus (Hitachi High-Technologies Corporation, Tokyo, Japan) operated in a high vacuum mode at a 15-kV accelerating voltage. As it was mentioned, no special sample preparation was made before analysis. Just a drop of the sample aqueous solution (3 µL) containing microgels, lipid vesicles or lipobeads is deposited either directly on an Al-mount or a carbon conductive tab preliminary adhered on the mounting stub. After a few minutes, the excess solution is absorbed with a strip of the filter paper and the mount is transferred to the imaging chamber of the microscope without further drying or coating. When a charge-up phenomenon is observed in the Conductor or Standard (high-vacuum) modes, the Charge-up Reduction mode (low-vacuum functionality) is applied. The images are taken at different magnifications to characterize the whole range of the sizes, morphologies, and structures of the microparticles studied.

#### 4.7.4. Atomic Force Microscopy of Lipobeads

AFM measurements is carried out in tapping mode in free air at room temperature (25 °C) with a Multimode NanoScope IIIa (Digital Instruments, Santa Barbara, CA, USA). Typically, 4 µL of particles’ dispersion in water is deposited on fresh mica cleaved by pressing an adhesive tape against the top mica surface and peeling off the tape. The sample on the mica surface is dried under nitrogen flow for a few minutes before imaging.

## Figures and Tables

**Figure 8:** Confocal and electron microscopy images of the lipobeads resulted from mixing PNIPA-FA hydrogel microspheres and lipidic formulation #1 in Table 1 before (**A**) and after (**B**) sonication, respectively. The Green images originate from fluorescein-*o*-acrylate (FA) covalently attached to the PNIPA network. The Red images originate from rhodamine B covalently attached to the heads of phospholipid (RhodB-PE). For the CLSM images, an aliquot of the lipobeads’ suspension was placed onto the depression slide. For the SEM images, the wet lipobeads from the same batch were deposited on a carbon tab adhered on the SEM mount.

**Table:** 

	Confocal Scanning Microscopy			Scanning Electron Microscopy		
	Bright Field	Hydrogel Core	Lipid Bilayer	×2000	×5000	×10,000
**A** before sonication	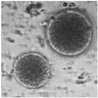	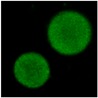	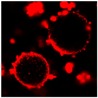	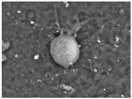	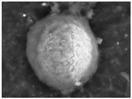	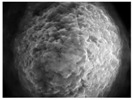
**B** after sonication	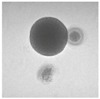	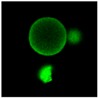	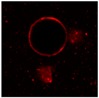	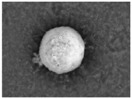	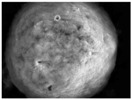	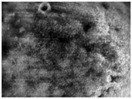

**Table 1:** Structure and morphology of giant multilamellar vesicles for different compositions and preparation methods of lipid formulations as imaged by the high vacuum SEM.

Formulation Number Composition Method	Three Types of Vesicles		
	Small: <5 μm	Medium: 5–10 μm	Large: >10 μm
1 HSPC/Chol/RhodB-PE Injection	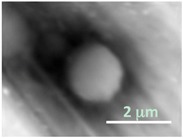	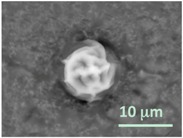	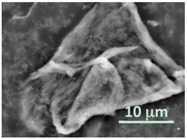
2 HSPC/RhodB-PE Injection	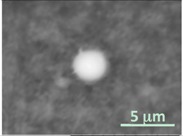	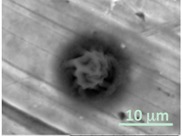	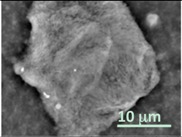
3 EPC/Chol/RhodB-PE Injection	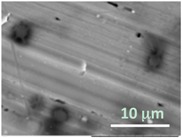	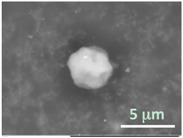	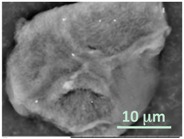
4 EPC/RhodB-PE Injection	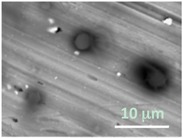	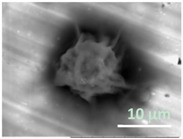	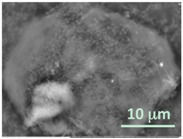
5 EPC/Chol/RhodB-PE Gentle Hydration	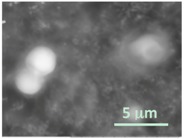	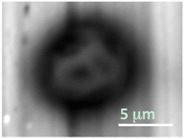	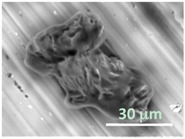
6 EPC/RhodB-PE Gentle Hydration	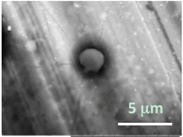	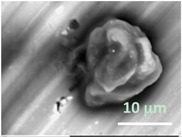	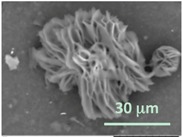

**Table 2:** Structure and morphology of giant lipobeads prepared by incubation of giant multilamellar vesicles (GMVs) and microgels as imaged by the high vacuum SEM at three different magnifications (the scale bars correspond to all images in the same column).

Formulation Number Composition Method	Giant Lipobeads		
	×2000	×5000	×10,000
1 HSPC/Chol/RhodB-PE Injection	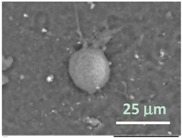	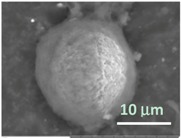	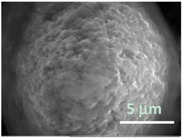
2 HSPC/RhodB-PE Injection	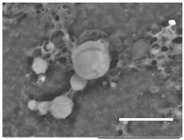	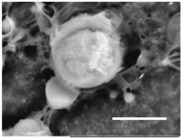	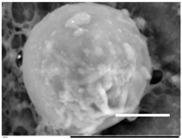
3 EPC/Chol/RhodB-PE Injection	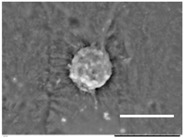	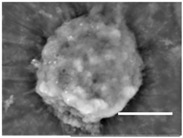	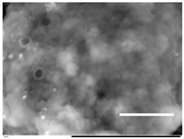
4 EPC/RhodB-PE Injection	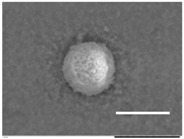	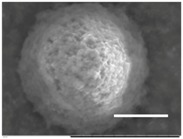	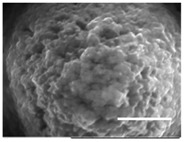
5 EPC/Chol/RhodB-PE Gentle Hydration	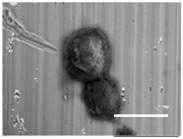	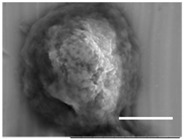	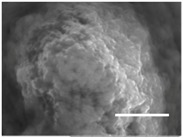
6 EPC/RhodB-PE Gentle Hydration	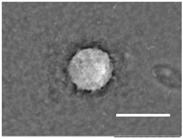	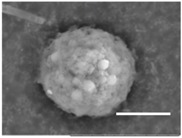	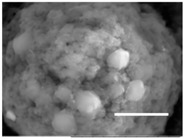

**Table 3:** Bulk fluorescent hydrogels: composition of hydrogel forming solutions and conditions of polymerization.

Type of Polymerization	Solvent	HGFS					*T* °C	Polymerization Time
		NIPA	MBA	DEAP	APS	TEMED		
UV	Water	0.44 M	65 mM	5 mM	–	–	25	1 h
Thermal	Water	0.88 M	0.13 M	–	4.4 mM	13.4 mM	60	
	DMSO							

“–“ Means that this component does not present in the HGFS.
